# Impact of Different Metal Artifact Reduction Techniques on Attenuation Correction of Normal Organs in ^18^F-FDG-PET/CT

**DOI:** 10.3390/diagnostics12020375

**Published:** 2022-02-01

**Authors:** Janna Morawitz, Ole Martin, Johannes Boos, Lino M. Sawicki, Katrin Wingendorf, Martin Sedlmair, Eduards Mamlins, Christina Antke, Gerald Antoch, Benedikt M. Schaarschmidt

**Affiliations:** 1 Department of Diagnostic and Interventional Radiology, Medical Faculty, University Dusseldorf, D-40225 Dusseldorf, Germany; o.f.martin@web.de (O.M.); boos@radiologie-muenster.de (J.B.); lino.sawicki@radiologie-wesel.de (L.M.S.); katrin@kkkw.de (K.W.); antoch@med.uni-duesseldorf.de (G.A.); 2 Department of Computed Tomography, Siemens Healthineers GmbH, D-91301 Forchheim, Germany; martin.sedlmair@siemens.com; 3 Department of Nuclear Medicine, Medical Faculty, University Dusseldorf, D-40225 Dusseldorf, Germany; eduards.mamlins@med.uni-duesseldorf.de (E.M.); christina.antke@med.uni-duesseldorf.de (C.A.); 4 Institute of Diagnostic and Interventional Radiology and Neuroradiology, University Hospital Essen, D-45147 Essen, Germany; benedikt.schaarschmidt@uk-essen.de

**Keywords:** positron emission tomography/computed tomography, artifacts, image processing, computer-assisted

## Abstract

Purpose: To evaluate the impact of different metal artifact reduction algorithms on Hounsfield units (HU) and the standardized uptake value (SUV) in normal organs in patients with different metal implants. Methods: This study prospectively included 66 patients (mean age of 66.02 ± 13.1 years) with 87 different metal implants. CT image reconstructions were performed using weighted filtered back projection (WFBP) as the standard method, metal artifact reduction in image space (MARIS), and an iterative metal artifacts reduction (iMAR) algorithm for large implants. These datasets were used for PET attenuation correction. HU and SUV measurements were performed in nine predefined anatomical locations: liver, lower lung lobes, descending aorta, thoracic vertebral body, autochthonous back muscles, pectoral muscles, and internal jugular vein. Differences between HU and SUV measurements were compared using paired *t*-tests. The significance level was determined as *p* = 0.017 using Bonferroni correction. Results: No significant differences were observed between reconstructed images using iMAR and WFBP concerning HU and SUV measurements in liver (HU: *p* = 0.055; SUVmax: *p* = 0.586), lung (HU: *p* = 0.276; SUVmax: *p* = 1.0 for the right side and HU: *p* = 0.630; SUVmax: *p* = 0.109 for the left side), descending aorta (HU: *p* = 0.333; SUVmax: *p* = 0.083), thoracic vertebral body (HU: *p* = 0.725; SUVmax: *p* = 0.392), autochthonous back muscles (HU: *p* = 0.281; SUVmax: *p* = 0.839), pectoral muscles (HU: *p* = 0.481; SUVmax: *p* = 0.277 for the right side and HU: *p* = 0.313; SUVmax: *p* = 0.859 for the left side), or the internal jugular vein (HU: *p* = 0.343; SUVmax: *p* = 0.194). Conclusion: Metal artifact reduction algorithms such as iMAR do not alter the data information of normal organs not affected by artifacts.

## 1. Introduction

Although huge technical improvements have been made in reducing artifacts in computed tomography (CT) in the last decades, especially metal artifacts still degrade the diagnostic value of CT images. Particularly, in positron emission tomography/computed tomography (PET/CT), CT artifacts are a major problem. Apart from diagnostic problems caused by image quality degradation in CT, quantification of tracer uptake can be falsified. As attenuation correction in PET/CT is based on CT data, a dark band artifact caused by a metal implant can lead to an underestimation of attenuation and consecutively falsely high standardized uptake value (SUV) measurements in attenuation-corrected images, while a bright band artifact caused by a metal implant can lead to an overestimation of attenuation, resulting in falsely low SUV measurements [1]. Therefore, the use of modern metal artifact reduction techniques has become of considerate interest in recent PET/CT research [2,3,4,5].

As beam hardening, scatter, photon starvation, noise, and edge effects are the main cause of metal artifacts, different strategies are used to minimize them: (1) modifying standard acquisition and reconstruction, (2) application of dual-energy CT, and (3) modifying projection data and/or image data [6]. Increasing kVp and mAs, and reducing collimation and the use of a soft reconstruction kernel are useful to modify standard acquisition but can also increase the radiation dose [7]. Dual-energy computed tomography (DECT) can reduce artifacts by creating virtual monocromatic images using two different photon spectra but require dedicated hardware and scanning protocols [8]. The most widely used strategy to reduce metal artifacts is applying Metal Artifact Reduction (MAR) software. Commercial software such as MAR in space (MARIS) and iterative MAR (iMAR) are sinogram inpainting techniques that incorporate high-frequency data from standard weighted filtered back projection (WFBP) reconstructions to reduce metal artifacts [9]. These algorithms replace corrupted projections caused by metal with interpolation from neighboring uncorrupted projections [10]. Due to the difference of these technical approaches, the use of different metal artifact reduction algorithms can have impact on SUV measurements [11].

As there is an increasing number of clinical applications for PET/CT as well as a further increase of metal implants as HIP-implants or pacemakers in an aging society, it is essential to improve CT image quality and free attenuation-corrected PET images from quantification errors [12,13]. Still, it is of utmost importance that the CT and PET information of regions that are not affected by artifacts are not altered by the applied metal artifact reduction technique. This is of particular interest if PET/CT imaging is used for follow-up imaging in oncological diseases, especially when relative response assessment criteria rely on reference measurements of the mediastinal/jugular blood pool or the liver, such as the Deauville criteria in lymphoma or the Hopkins criteria in head and neck cancer imaging [14,15]. Therefore, the aim of this study was to investigate whether different metal artifact reconstruction algorithms have an impact on the tracer uptake of normal organs in attenuation-corrected PET images in PET/CT.

## 2. Materials and Methods

### 2.1. Patients

Patients that underwent a clinically indicated PET/CT with metal implants in different anatomical localizations were prospectively included in this study. This study was approved by the institutional review board and written informed consent was obtained from all patients prior to the examination.

Patients fasted for 6 h prior to the examination and blood glucose level was verified to be below 150 mg/dL at injection time. ^18^F-fluordesoxyglucose (^18^F-FDG) was used as the tracer in all patients.

### 2.2. Data Acquisition

^18^F-FDG PET/CT was acquired on a Biograph mCT PET/CT scanner (Siemens Healthineers, Erlangen, Germany) 63.7 ± 7.6 min after tracer injection with a mean activity of 225 ± 30 MBq. In 34.8% (23/66) of all patients, a whole-body PET/CT was acquired from the head to the feet. In 65.2% (43/66) of the patients, a PET/CT from the body trunk was acquired from the base of the skull to the proximal femora. In total, 80.3% (53/66) of the patients underwent PET/CT 70 s after intravenous administration of 100 mL of an iodinated contrast agent (Accupaque 300; GE Healthcare Buchler GmbH & Co. KG, Braunschweig, Germany). In total, 19.7% (13/66) of the patients underwent low-dose CT without administration of the contrast agent. CT examinations were performed with automated tube current modulation (CareDose 4D, Siemens Healthineers, Erlangen, Germany) with a reference tube current time product of 190 mAs, as specified in our standard protocol for whole-body PET/CT examinations. With a reference value of 120 kVp, the automated tube voltage selection was used (CarekV, Siemens Healthineers, Erlangen, Germany). CT data were acquired with a 0.5 s rotation time, 32 × 1.2 mm collimation, and a pitch of 0.8. PET data were acquired for 3 min per bed position.

### 2.3. CT Image Reconstruction

CT raw data were reconstructed on a workstation using a dedicated reconstruction software (ReconCT v. 13.8.2.0, Siemens Healthineers, Erlangen, Germany). Reconstructions were performed using weighted filtered back projection (WFBP) as the standard method, MAR in image space (MARIS), and an iterative metal artifacts reduction (iMAR) 2D algorithm for large implants (called “hip implant”, hip). The reconstructed WFBP images as well as the images with MAR algorithms were reconstructed in axial orientation, with a slice thickness of 5 mm and a 2 mm increment using a medium-smooth kernel (B30f) and a matrix size of 512 × 512.

### 2.4. PET Reconstruction

Attenuation-corrected PET images were reconstructed using WFBP, MARIS, and iMAR-hip CT datasets using ordered subset expectation maximization with four iterations and eight subsets. The slice thickness was matched to the CT images. A Gaussian filter kernel with a full width at the half-maximum of 2.0 mm was used for post-reconstruction filtering, with a transaxial matrix size of 200 × 200.

### 2.5. Image Analysis

**Figure 1:**
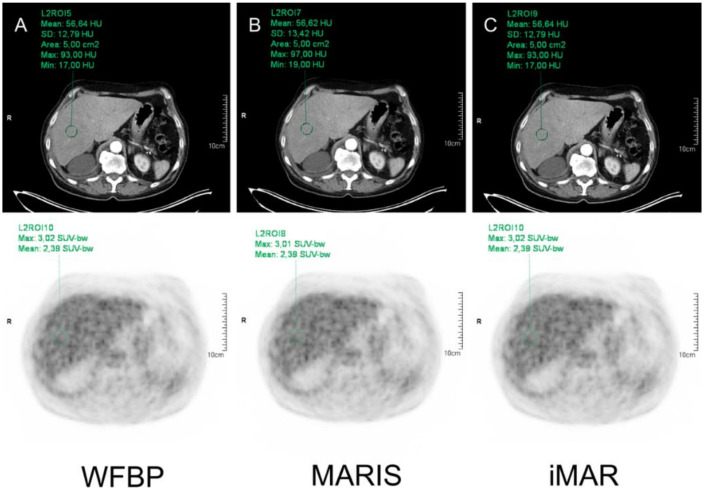
Example of HU and SUV measurement in the liver in WFBP (**A**), MARIS (**B**), and iMAR (**C**).

CT Images using WFBP, MARIS, and iMAR-hip as algorithms were investigated for HU analysis. For SUV analysis, PET images based on the three CT reconstructions were used. In WFBP reconstruction, a circular region of interest (ROI) was placed in the respective organ and was automatically copied to MARIS and iMAR-hip reconstruction as well as to all three PET reconstructions (Figure 1).

**Figure 2:**
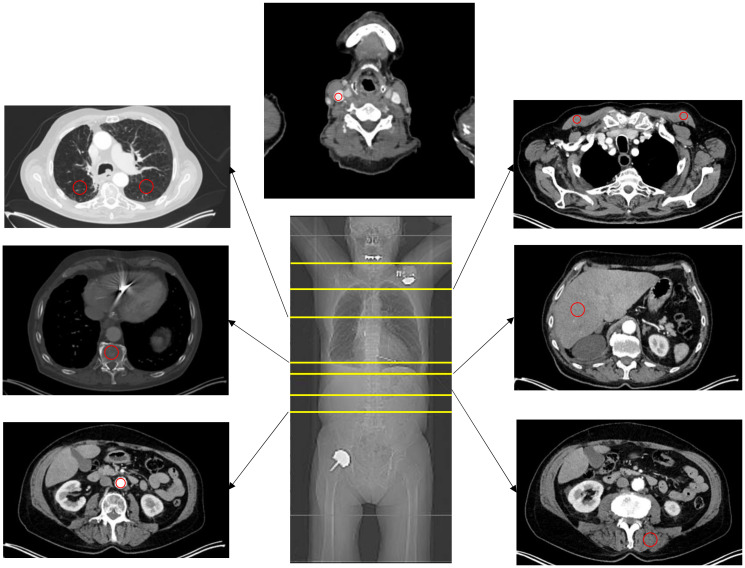
Overview of different measurement localizations. The red circle indicates the localization of the measurements.

Average Hounsfield Unit (HU) values in the CT images as well as SUVmean and SUVmax values in PET images were measured for each ROI. Measurements were conducted in the liver (segment 6), lung (right and left lower lobe, segment 6), descending aorta, thoracic vertebral body 12, autochthonous back muscles, pectoral muscles, and internal jugular vein (Figure 2). The respective ROI was placed outside of artifacts and in morphologically inconspicuous areas.

### 2.6. Statistical Analysis

Statistical analysis was performed using SPSS Statistics v 26 (IBM, Armonk, NY, USA). In WFBP, MARIS, and iMAR-hip, mean values and standard deviation were calculated in CT as well as SUVmean and SUVmax in PET reconstructions in all predefined normal organ areas. A paired *t*-test was used to compare differences between HU and SUV measurements in WFBP, MARIS, and iMAR-hip in normal organs. Due to multiple testing, Bonferroni correction was used and *p* = 0.017 was considered as statistically significant.

## 3. Results

### 3.1. Patient Population

Sixty-six patients (28 female and 38 male; mean age of 66.02 ± 13.1 years) were prospectively included in the study between March 2017 and August 2017. The involved patients had eighty-seven metal implants, which included port catheters (*n* = 29), hip implants (*n* = 18), knee implants (*n* = 12), pacemakers (*n* = 8), dental implants/fillings (*n* = 7), shoulder implants (*n* = 3), spine implants (*n* = 3), humerus implants (*n* = 3), femoral nails (*n* = 2), a tracheostoma (*n* = 1), and a ureteral stent (*n* = 1).

### 3.2. HU Measurements

In WFBP, HU measurements for liver were 82.72 ± 26.95 HU, whereas measurements in MARIS and iMAR were 82.80 ± 26.94 HU and 82.69 ± 26.92 HU, respectively. For the right lung and left lung, HU measurements in WFBP, MARIS, and iMAR were −685.79 ± 103.02 HU vs. −684.41 ± 104.38 HU vs. −685.94 ± 103.12 HU (right lung) and −679.63 ± 95.31 HU in all three reconstruction algorithms (left lung). Thoracal vertebral body 12 showed HU measurements of 157.06 ± 67.58 HU vs. 157.38 ± 64.33 HU vs. 157.08 ± 67.53 HU in WFBP, MARIS, and iMAR, and 119.91 ± 56.20 HU vs. 119.75 ± 56.11 HU vs. 119.68 ± 56.13 HU for the descending aorta. In WFBP, the HU measurements for autochthonous back muscles were 43.79 ± 17.66 HU, whereas measurements for MARIS and iMAR were 44.05 ± 17.58 HU and 44.93 ± 15.06 HU, respectively.

For the right and left pectoral muscle, HU measurements in WFBP, MARIS, and iMAR were 51.31 ± 13.97 HU vs. 51.14 ± 13.87 HU vs. 51.01 ± 13.06 HU (right) and 53.57 ± 14.42 HU vs. 53.38 ± 14.66 HU vs. 52.05 ± 10.16 HU (left). For the jugular vein, HU measurements were 152.85 ± 41.89 HU vs. 152.93 ± 41.83 HU vs. 153.34 ± 45.30 HU.

Compared to WFBP, the metal artifact reduction algorithms MARIS and iMAR showed no significant impact on HU measurements in all investigated organs (Table 1).

### 3.3. SUV Measurements

The SUVmax in the liver was 3.05 ± 0.61 in WFBP and iMAR, while measurements in MARIS showed a SUVmax of 3.04 ± 0.61. For the right lung, SUVmax was 0.93 ± 0.37 in WFBP as well as in iMAR, and 0.92 ± 0.37 in MARIS, while the left side showed a SUVmax of 0.91 ± 0.38 in all three reconstruction algorithms. SUVmax in thoracal vertebral body 12 showed values of 2.56 ± 1.09 in WFBP and MARIS, while it was 2.54 ± 1.11 for iMAR. In the descending aorta, SUVmax was 2.28 ± 0.49 in WFBP and iMAR, whereas MARIS showed a value of 2.27 ± 0.52. The autochthonous back muscles showed a SUVmax of 0.91 ± 0.28 in WFBP, 0.89 ± 0.20 in MARIS, and 0.91 ± 0.25 in iMAR. For the pectoral muscles, SUVmax was 0.79 ± 0.28 in WFBP and iMAR, and 0.78 ± 0.28 in MARIS (right side), while the left side showed a SUVmax of 0.78 ± 0.24 in WFBP and iMAR, and a SUVmax of 0.78 ± 0.23 in MARIS. In the jugular vein, SUVmax was 1.85 ± 0.39 in WFBP and iMAR, whereas measurements in MARIS showed a value of 1.84 ± 0.39.

Consecutively, no significant differences were observed in all analyzed locations (Table 1).

## 4. Discussion

Our study demonstrates that MARIS and iMAR do not have an impact on HU or SUV measurements in normal organs if the ROI is placed outside of artifacts. Therefore, these algorithms can be reliably applied in PET/CT datasets in patients with metal implants to improve image quality, without altering image information in normal organs.

Metal artifact reduction algorithms are known to improve the image quality of CT images and to reduce both dark and bright band artifacts without the need of dedicated devices or dedicated scanning protocols. As dark and bright band artifacts can lead to an under or overestimation of attenuation, respectively, attenuation correction based on CT data, such as in current PET/CT scanners, could be severely impaired. Thus, SUV measurement could be falsified. This has to be considered as a serious problem as in oncology, quantification of SUV measurements is used to assess treatment response [16,17]. As a comparison of SUV measurements considered as difficult, scores comparing the pathological tracer uptake to normal organ values, such as the Lugano classification or the Hopkins criteria, are mainly used in clinical practice [15,18,19]. Therefore, it is of utmost importance to investigate the impact of MAR algorithms on normal organ values before using this technique for CT-based attenuation correction. All tested reconstruction algorithms did not have any significant impact on HU or SUV measurements in normal organs. This is of added value to previous results, showing that iMAR improves the delineation of anatomical structures in the vicinity of metal implants in CT [2,20]. Martin et al. could furthermore show that iMAR improves PET image attenuation correction and provide more reliable, quantitative SUV measurements adjacent to large metal implants as well as port chambers [11,21]. Furthermore, Kennedy et al. showed that artifact reduction techniques can improve target-to-background ratios in the vicinity of metal implants [22].

While several studies have shown the value of iterative metal artifact reduction techniques in phantom as well as patient-studies and its clear benefits, new “MAR-induced” artifacts can be challenging as they can mimic pathologic processes in CT images, especially in the vicinity of metal implants; for example, such as peri-hardware lucency or material failure [10,23]. However, MAR-induced artifacts have not been described for areas that are not affected by artifacts. As the impact of these artifacts on PET quantification has to be considered low, their importance in PET/CT should not be overestimated. However, they should be kept in mind when interpreting hybrid imaging data.

Besides iMAR, DECT is a possible alternative to reduce metal artifacts [10]. While MAR software only uses available single-source CT data, DECT requires the acquisition of CT images at two different energy levels during examination, thus necessitating dedicated dual-source CT scanners which are not available in current-generation PET/CT scanners. While the acquisition of sequential CT scans at different energy levels is possible at current-generation PET/CT, this leads to a considerate increase in radiation exposure and should be therefore used in selected cases only. However, an initial phantom study suggests that iMAR-corrected CT images might provide a superior attenuation correction compared to dual-energy CT images [24].

Our study has some limitations. We did not compare different MAR algorithms regarding attenuation correction in normal organs, as different algorithms can be useful, because, depending on the type and material of the implant, different iMAR algorithms show different suitability [25]. With iMAR-hip, we used the algorithm with the strongest artifact reduction to provoke potential artifacts in normal organ measurements. In addition, this is a single-center study, which is why statements can only be made about the PET/CT scanner used here (Biograph mCT PET/CT scanner by Siemens Healthineers, Erlangen, Germany). Further prospective studies are necessary to verify this for other devices used in the market. Furthermore, we did not compare the dual-energy artifact reduction technique considering in current-generation PET/CT scanners, dual-energy scanning can only be performed by sequential scanning, which is prone to motion artifacts and leads to an increased radiation exposure [26]. Moreover, we did not perform an analysis on the pathological lesions in this study as this topic has already been investigated before [2]. Only ^18^F-FDG was used as a tracer, as this is the most important tracer in oncological hybrid imaging [27].

In conclusion, metal artifact reduction algorithms such as iMAR are a reliable method for the Biograph mCT PET/CT scanner, which does not alter information in normal organs that are not affected by artifacts.

## Figures and Tables

**Table 1:** Values for HU and SUV measurements in different normal organs in WFBP, MARIS, and iMAR reconstruction, and the respective *p*-values.

		HU			SUVmax			SUVmean		
		WFBP	MARIS	iMAR	WFBP	MARIS	iMAR	WFBP	MARIS	iMAR
**Liver**		82.72 ± 26.95	82.80 ± 26.94	82.69 ± 26.92	3.05 ± 0.61	3.04 ± 0.61	3.05 ± 0.61	2.41 ± 0.46	2.40 ± 0.46	2.41 ± 0.46
*p*-value	WFBP vs. MARIS	0.295			0.095			0.353		
	WFBP vs. iMAR	0.055			0.568			0.159		
	MARIS vs. iMAR	0.157			0.084			0.278		
**Right lung**		−685.79 ± 103.02	−684.41 ± 104.38	−685.94 ± 103.12	0.93 ± 0.37	0.92 ± 0.37	0.93 ± 0.37	0.69 ± 0.27	0.69 ± 0.27	0.69 ± 0.27
*p*-value	WFBP vs. MARIS	0.229			0.603			0.536		
	WFBP vs. iMAR	0.276			1.0			0.568		
	MARIS vs. iMAR	0.179			0.602			0.480		
**Left lung**		−679.63 ± 95.31	−679.63 ± 95.31	−679.63 ± 95.31	0.91 ± 0.38	0.91 ± 0.38	0.91 ± 0.38	0.67 ± 0.26	0.67 ± 0.26	0.67 ± 0.26
*p*-value	WFBP vs. MARIS	1.0			0.049			0.418		
	WFBP vs. iMAR	0.630			0.109			0.045		
	MARIS vs. iMAR	0.629			0.251			0.159		
**Vertebral body**		157.06 ± 67.58	157.38 ± 64.33	157.08 ± 67.53	2.56 ± 1.09	2.56 ± 1.09	2.54 ± 1.11	1.97 ± 0.73	1.96 ± 0.72	1.96 ± 0.72
*p*-value	WFBP vs. MARIS	0.835			0.871			0.412		
	WFBP vs. iMAR	0.725			0.392			0.515		
	MARIS vs. iMAR	0.837			0.383			0.490		
**Descending aorta**		119.91 ± 56.20	119.75 ± 56.11	119.68 ± 56.13	2.28 ± 0.49	2.27 ± 0.52	2.28 ± 0.49	1.86 ± 0.37	1.86 ± 0.38	1.86 ± 0.37
*p*-value	WFBP vs. MARIS	0.038			0.241			0.080		
	WFBP vs. iMAR	0.333			0.083			0.159		
	MARIS vs. iMAR	0.801			0.229			0.109		
**Autochthonous back muscles**		43.79 ± 17.66	44.05 ± 17.58	44.93 ± 15.06	0.91 ± 0.28	0.89 ± 0.20	0.91 ± 0.25	0.72 ± 0.20	0.70 ± 0.13	0.71 ± 0.19
*p*-value	WFBP vs. MARIS	0.164			0.316			0.342		
	WFBP vs. iMAR	0.281			0.839			0.937		
	MARIS vs. iMAR	0.402			0.306			0.322		
**Pectoral muscle (right)**		51.31 ± 13.97	51.14 ± 13.87	51.01 ± 13.06	0.79 ± 0.28	0.78 ± 0.28	0.79 ± 0.28	0.66 ± 0.26	0.66 ± 0.26	0.66 ± 0.26
*p*-value	WFBP vs. MARIS	0.055			0.097			0.117		
	WFBP vs. iMAR	0.481			0.277			0.410		
	MARIS vs. iMAR	0.767			0.047			0.070		
**Pectoral muscle (left)**		53.57 ± 14.42	53.38 ± 14.66	52.05 ± 10.16	0.78 ± 0.24	0.78 ± 0.23	0.78 ± 0.24	0.65 ± 0.20	0.65 ± 0.20	0.65 ± 0.20
*p*-value	WFBP vs. MARIS	0.184			0.054			0.223		
	WFBP vs. iMAR	0.313			0.859			0.260		
	MARIS vs. iMAR	0.393			0.029			0.028		
**Jugular vein**		152.85 ± 41.89	152.93 ± 41.83	153.34 ± 45.30	1.85 ± 0.39	1.84 ± 0.39	1.85 ± 0.39	1.63 ± 0.37	1.64 ± 0.39	1.64 ± 0.38
*p*-value	WFBP vs. MARIS	0.435			0.512			0.169		
	WFBP vs. iMAR	0.343			0.194			0.332		
	MARIS vs. iMAR	0.362			0.251			0.328		

HU: Hounsfield unit; MARIS: metal artifact reduction in space; SUV: standardized uptake value; WFBP: weighted filtered back projection; and iMAR: iterative metal artifact reduction.

## Data Availability

The datasets generated and/or analyzed during the current study are not publicly available due to continuous research on this topic but are available from the corresponding author upon reasonable request.
